# Drought Response in Rice: The miRNA Story

**DOI:** 10.3390/ijms20153766

**Published:** 2019-08-01

**Authors:** Kalaivani Nadarajah, Ilakiya Sharanee Kumar

**Affiliations:** School of Environmental and Natural Resource Sciences, Faculty of Science and Technology, Universiti Kebangsaan Malaysia, 43600 UKM Bangi, Malaysia

**Keywords:** miRNA, drought, rice, hormone, osmoregulation, antioxidant defense, senescence, growth and development

## Abstract

As a semi-aquatic plant, rice requires water for proper growth, development, and orientation of physiological processes. Stress is induced at the cellular and molecular level when rice is exposed to drought or periods of low water availability. Plants have existing defense mechanisms *in planta* that respond to stress. In this review we examine the role played by miRNAs in the regulation and control of drought stress in rice through a summary of molecular studies conducted on miRNAs with emphasis on their contribution to drought regulatory networks in comparison to other plant systems. The interaction between miRNAs, target genes, transcription factors and their respective roles in drought-induced stresses is elaborated. The cross talk involved in controlling drought stress responses through the up and down regulation of targets encoding regulatory and functional proteins is highlighted. The information contained herein can further be explored to identify targets for crop improvement in the future.

## 1. Introduction

miRNAs function in controlling expression levels of target genes regulating physiological and developmental processes in plants, animals, and microbes [1,2]. They are able to regulate multiple targets and thence act as master regulators of processes such as growth, photosynthesis, respiration, and response to biotic and environmental stresses. As a master switch, miRNA may be exploited to control or improve agronomic traits such as resistance and tolerance towards stresses [3,4,5]. The miRNA biogenesis begins with the transcription of miRNA genes through RNA polymerase II that generates primary miRNA transcripts via DICER like 1 (DCL1). DCL1 then further processes precursor miRNAs to miRNA duplexes in the nuclei. The miRNA is then transferred to the cytoplasm where through ARGONAUTE (AGO), it regulates the cleavage of mRNA [6,7]. As part of the regulatory network, miRNAs work closely with target genes and transcription factors. The interaction between these components are important for the regulation of plant developmental processes and is absolutely essential in moderating gene expression in plants [8]. The interaction of these miRNAs, their target genes, and the transcription factors therein differ between organisms and exhibit differential expression spatially and temporally. As part of the regulatory network, miRNAs work closely with target genes and various transcription factors such as APETALA 2 (*AP2*), *GRF* (Growth-Regulating Factor), *NAC* (*NAM*, *ATAF1*, and *CUC2*), *NF-Y* (Nuclear transcription factor Y), *MYB*, *TCP* (TEOSINTE BRANCHED/CYCLOIDEA/PCF), *SPL* (SQUAMOSA-promoter binding transcription factor) and *WRKY* [9]. The overlap between miRNAs and their targets in multiple processes show that there is cross talk between various physiological and developmental processes in plants [10,11]. From the studies conducted in various plant systems, one can conclude that miRNAs are regulated by multiple factors and are dynamic [12,13].

Rice is a staple diet of most Asian countries. Various countries grow rice for local consumption, export, and income. With the growing population, the demand made on the supply of rice is high. Therefore, breeding programs are directed towards increasing yield in response to both biotic and abiotic stresses [14,15]. One such stress that affects yield is drought. It is therefore important to understand how drought affects rice and its yield. Drought-prone as well as poorly irrigated regions require low water utilization efficiency in their crops. The lack of water can affect development and physiological processes within the plant and result in detrimental outcomes. It is therefore important to identify factors that regulate against drought and to find key components, such as miRNAs that may be used in the development of drought resistant plants. Plants have evolved several mechanisms that enable them to withstand stresses. These mechanisms include root elongation, osmotic stress regulation, regulation of photosynthesis and respiration, senescence, antioxidative stress modulation, and various hormonal fine-tuning of physiological and developmental processes within the plant. Studies have shown that miRNAs are important modulators of drought tolerance in plants where it influences the cleavage of several drought responsive genes and thence inhibits their translation. The regulatory information derived from these components control the networking between miRNA and their target genes [16]. However, to date, the information on miRNAs, target genes and the cross talk between pathways is not well understood in plants, especially in rice. Therefore, further systematic elucidation is required to understand the regulatory networks and useful strategies for crop improvement [13,17,18,19].

In this review we look into the role of miRNA in regulating drought stress in rice. The summary of information obtained from a systematic review is useful in understanding the role of miRNAs in drought response and to identify key modulators that may be targeted for rice crop improvement in the near future. The miRNAs identified in rice will also be compared with reports from other plant systems to observe the similarities and differences in the mechanisms of regulation in drought. By understanding the contribution of each miRNA in influencing their target genes and processes, we are better equipped to understand the complexities involved in managing drought. Some of these miRNAs while mitigating stress are also responsible for yield elevation. The following sections will elucidate their involvement in hormonal, developmental, and stress management during drought in rice and compare them with other plant systems. Key miRNAs for further functional analysis will also be identified.

## 2. Hormonal Regulation of Drought

### 2.1. Abscisic Acid (ABA) in Drought Stress Signaling

Abscisic acid (ABA) plays a dominant role in drought regulation by addressing water deficit and mediating stress response through the activation of appropriate genes and stomatal movement [20,21]. In plants, abiotic stresses are both regulated by ABA dependent and non-dependent pathways [22], which are controlled by their respective regulatory elements [23]. However, it was not until the isolation of ABA hypersensitive mutant, that the role of miRNA in regulating ABA responses was recognized. Through mutant analysis, several genes that are involved in miRNA biogenesis pathways such as *DCL1* (Dicer-like 1), *HASTY*, *HEN1* (Small RNA 2′-O-methyltransferase), *HYL1* (double-stranded RNA-binding protein 1), and *SE* (Serrate RNA effector molecule) were identified. Mutants of *dcl1* and *hen1* were shown to be sensitive to ABA during germination while the *se* and *hasty* mutants were sensitive to high osmosis and salt stresses [24]. Collectively we note that the members of the miRNA biogenesis may control drought responses in plants through various cellular and molecular processes that are controlled via a series of feedback regulations [25,26].

**Figure 1:**
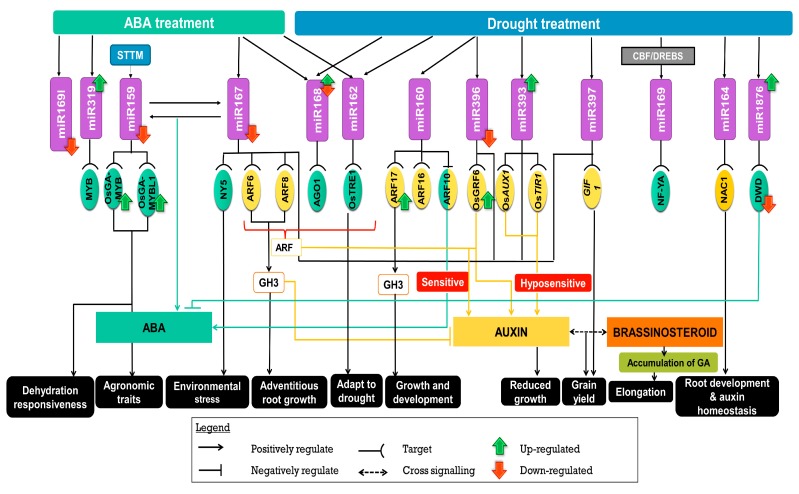
The regulation of microRNA under drought stress by phytohormones in rice. The legend above provides elaboration on the functions depicted by each arrow. The above miRNAs were identified post treatment with ABA and drought. Their up or down regulation resulted in a positive or negative effect on target transcription factors (auxin response factor (ARF), NAC, MYB, Nuclear factor Y (NFY), and DDB1 binding WD40 (DWD)). These interactions resulted in either an increase or decrease in the expression of drought stress related genes such as GH3. Hormones such as auxin, Abscisic acid (ABA), gibberellin (GA), and brassinosteroid (BR) play an important role in drought response. The processes affected by the interaction between the miRNA-Transcription Factor-gene complex is elaborated within the text.

Transcription factors are key regulators of various processes in plants. MYB is implicated in several studies as a transcription factor that regulates dehydration responsiveness in plants [27]. ABA, through miRNAs, regulates genes that may aid in drought tolerance. Zhao et al. (2017) demonstrated that Short Tandem Target Mimic (STTM) suppresses miR159 expression, and subsequently increased the expression of two target genes, OsGAMYB and OsGAMYBL1 resulting in plants with short stature and smaller organs [28]. Further in a study conducted by Sunkar and Zhu (2007), miR159 was reported to be regulated by ABA in rice [29]. On the other hand, following inoculation with a root endophytic fungus *Piriformospora indica*, MYB targeting of miR159 was significantly induced in rice under drought [30]. In Arabidopsis, ABA and drought treatment resulted in elevated levels of miR159 which in turn cleaves MYB33 and MYB101 transcripts [31]. Further when overexpressed, miR159 suppresses MYB33 leading to ABA hyposensitivity. DWD (DDB1 binding WD40) is another protein that has been implicated in ABA regulated drought response. While MYB positively regulates ABA signaling, DWD regulates ABA negatively [32]. However, the up regulation of mi1876 reduces DWD activity thus reducing DWD’s negative effect on the regulation of ABA in rice roots. Under drought conditions, miR1876 was differentially expressed in rice [33]. However, miR1876 was not reported in *Arabidopsis thaliana* or other plant systems. Tian et al. (2015) through next generation sequencing (NGS) of *Osaba1*, an ABA deficient mutant, showed the influence of miR162b, an ABA responsive miRNA and its target gene *OsTRE1* (*trehalase 1*) in its adaptive role under drought stress and ABA treatment. The up regulation of *OsTRE1* caused the down regulation of miR162b which reduces trehalose accumulation leading to higher sensitivity to drought in rice [34] (Figure 1). Likewise, miR162 was down regulated in cotton but up regulated in plant systems such as Arabidopsis, barley (*Hordeum vulgare*), and maize (*Zea mays*) when subjected to drought [35].

Further, miR167 that targets auxin response factor (ARF) was shown to be down regulated by ABA in rice [36] and up regulated in Arabidopsis, wheat, and maize under water stress [37,38]. In maize, miR167d was down regulated under drought treatment, increasing the accumulation of Phospholipase D (PLD) which inhibits stress response [39]. PLD activation is vital for ABA signaling which regulates stomatal movement and responses in other plant systems including rice [40]. However, it is unknown whether PLD is targeted by miR167 in these plant systems. The ABRE motif containing miR168 is present in multiple species such as poplar, tobacco, Arabidopsis, maize, as well as in rice. Its AGO1 target is up regulated in Arabidopsis, in response to abiotic stimuli such as cold, high salinity, and drought [34] and down regulated in rice during drought [41,42]. NFY (Nuclear factor Y) directed ABA signaling was also implicated in drought stress tolerance in several of the following instances. For example, NFY5 is targeted by miR169a to withstand environmental stresses like drought [43,44]. NFY5A transcripts are highly expressed in stomatal guard cells and vascular tissues where they have control over the guard cell aperture. This transcript exerts control over several drought-responsive genes e.g., superoxide dismutase (*SOD*), glutathione transferase (*GT*) and peroxidases (*POD*) [44]. miR169a was down regulated in response to drought and ABA treatment resulting in induced levels of NFY5A transcript in Arabidopsis and tomato [45,46]. Further, miR169g was reported to be up regulated in roots and shoots of rice plants and is down regulated in Arabidopsis and Medicago under drought stress [47]. The expression of OsNF-YA7, a rice NFY transcription was induced in drought treatment but remained unchanged when treated with ABA proposing an ABA independent drought tolerance [48]. Previously, the miR169/NF-YA complex has been implicated in the regulation of plant stress responses [43]. Macovei et al. (2012) further noted that miR169 is regulated by CBF/DREBs (dehydration-responsive element) suggesting a role for miR169 in drought stress of rice [49,50]. The presence of DREs within the promoter of MIR169g enhances its role in drought [50]. Wheat miR169 on the other hand targets CCAAT-box transcription factor [51]. Interestingly, NF-YA is reported to bind to CCAAT-box transcription factor suggesting that the regulation of CCAAT-box transcription factor under drought is NF-YA independent in wheat. While miR169g was strongly up regulated, miR393 was transiently induced in rice [50]. When both miR167 and miR169 were down regulated, miR319 was up regulated in response to ABA in rice under normal conditions [36] (Figure 1). While no target was discovered for miR319 in the study conducted by Liu et al. (2009) [36], another study reported that TEOSINTE BRANCHED/CYCLOIDEA/PCF (TCP) could be a possible target [52]. miR319 that targets MYB family transcription factors are differentially expressed under drought stress in rice. gma-miR319c was up regulated during tillering and inflorescence-forming stages five days after water withholding (DAW) [53] but down regulated at six DAW [54]. Meanwhile, the overexpression of osa-miR319 that targets TCP TF in creeping bentgrass (*Agrostis stolonifera*) results in thicker and expanded leaves coated with increased leaf wax for drought tolerance [55]. We note that some miRNAs can interact or regulate other miRNAs to perform certain functions. For instance, MYB positively regulates ABA signaling through interaction with miR159 and miR167 in rice seedlings [9,56].

### 2.2. Auxin in Drought Stress Signaling

Water stress induced by drought results in inhibited growth and development allowing for diversion of energy and nutrients to enable plants to adapt to stress [57]. Auxin’s regulation and control of growth and development of drought stress in plants is modulated through several miRNA families. There are several transcription factor families such as auxin response factors (ARFs), NAC-domain and AGO1 [58] that regulate auxin-induced changes in tissues and organs. In tomato, miR160 was down regulated under drought condition whereas its target, ARF10 was significantly induced, implicating auxin’s role in stress responses [59]. The repression of ARF10 by miR160 in tomato has resulted in narrow leaf blades and low density of stomata. miR160 targets ARF10 and ARF16 while miR167 regulates ARF6 and ARF8 in rice [60,61]. Under drought stress, both miR160 and miR167 were down regulated in rice roots. In other crops such as wheat and cowpea, miR160 is found to be up regulated. As miR160 negatively regulates both ARF10 and ARF16, it is plausible that the down regulation of miR160 is crucial to increase root length by auxin under drought [62]. By negatively regulating ARF10, miR160 regulates ABA induced seed germination in Arabidopsis. Further, the miR167a-j regulates ARF6 and ARF8 which results in adventitious root formation in response to drought [63]. ARF6 and ARF8 negatively regulates auxin by controlling GH3 gene expression [64,65]. Mallory et al. (2005) presented that miR160 results in increased levels of ARF17 causing the accumulation of GH3 genes resulting in dramatic changes to growth and development in Arabidopsis [66,67]. NAC1 on the other hand is cleaved by miR164 which down regulates auxin signals related to lateral root development in tea plants under drought stress [68,69]. miR164 targeted NAC genes and was found to negatively regulate drought tolerance in rice [70]. Liu et al. (2008) in their study showed that miR160 and miR167 are involved in ABA responses in plants [71]. ABA down regulates miR167 in rice implicating a role in ARF induction and accumulation. Together with ABA, the auxin responsive ARFs jointly regulate drought stresses in rice, where the miRNAs in roots regulate auxin, nutrition, and stresses through feedback regulation between miR167 and ARFs [56]. miR160 on the other hand negatively regulates ARF10 resulting in ABA sensitivity which implies cross talk between both hormones [72]. The miR167-ARF8-GH3 pathway is highly conserved in rice [73] (Figure 1).

In Arabidopsis, miR393 is required for homeostasis of TIR1/AFB2 Auxin Receptor (*TAAR*) gene expression, which is important for various processes including drought tolerance [53]. In rice, it targets *OsTIR1* (*Transport inhibitor response 1*) and *OsAUX1* (*auxin transporter 1*) to restrict growth. Further, different miRNAs connected to auxin signaling have been shown to affect developmental activities such as tillering and branching. miR156 regulates yield (OsSPL14) while miR393a, miR396b, miR397, and miR167 promotes panicle branching and better grain yield [16,74,75]. The up regulation of miR397 in rice negatively regulates *OsLAC* (laccase) which is associated to brassinosteroids and grain yield increase [75]. miR397 was also induced under drought conditions in rice [28] and Arabidopsis where it targets the laccase genes [76]. Majority of the miRNAs above target GRF-interacting factor 1 (GIF1), which are responsible for cell proliferation and growth [35,77,78]. The suppression of miR396 up regulates auxin responsive genes, where OsGRF6 positively regulates auxin signaling in rice [16,79]. Contrary to the above, miR396 is up regulated in drought-stressed Arabidopsis [71] and tobacco [59]. Cross talk and co-expression network of miR396 and miR397 provides evidence that there is a network between brassinosteroid (BR) and auxin in regulating growth and yield in rice [80]. Besides BR, there is also the accumulation of gibberellin (GA) that is able to stimulate elongation through the interaction of miRNAs and target genes such as *SCR* (*Scarecrow*), *DELLA*, and *GRF*. BR cross talks with auxin and or GA to control yield in rice [81] (Figure 1).

## 3. The Role of miRNA in Growth and Development during Drought

Hormones largely regulate growth and development [82]. However, key players such as miR396 repress GRF activity [83,84] when overexpressed to enhance drought tolerance as well as leaf development in *Medicago truncatula* and Arabidopsis [36,47]. In rice, miR396 was down regulated under drought conditions and up regulated following inoculation with *P. indica*, resulting in down regulation of GRF. This lowered cell proliferation and growth which subsequently reduced transpiration for drought tolerance [30].

**Figure 2:**
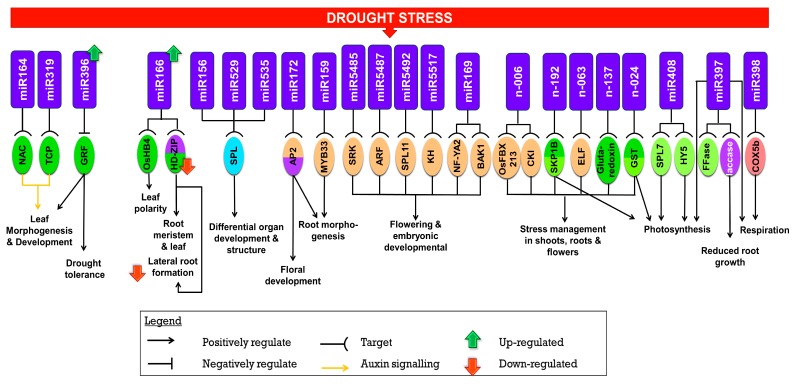
The regulation of microRNA under drought stress involving growth, development, photosynthesis and respiration. The legend above provides elaboration on the functions depicted by each arrow. The above miRNAs were identified under drought stress and their regulation resulted in a positive or negative effect on target transcription factors (ARF, NAC, GRF, HD-ZIP, SPL, MYB, NFY) or genes. These interactions resulted in either an increase or decrease in the expression of genes related to growth and development, photosynthesis, and respiration for better adaptation under drought. The interactions between the miRNAs-Transcription Factors-gene complex has been elaborated on within the text. The putative microRNAs (n-006, n-192, n-063, n-137, and n-024) are novel microRNAs from rice.

miR156 targets SPL that remains constant through embryogenic [85], trichome [86], flower, and fruit developmental stages [35]. Transcriptome studies in wild type and miR156 overexpressing rice lines showed a temporal change in expression of young to old leaves. Gene expression changes in these overexpressing lines resulted in rapid maturation and tillering of plants. miR156 was significantly induced in Arabidopsis and rice, where it negatively regulates SPL9 to increase abiotic stress tolerance such as salinity and drought [87]. In another study, the overexpression of miR156 up regulated OsSPL14 in rice which is associated with an ideal plant architecture where a point mutation of this gene reduces the number of tillers, increases yield, and contributes to resistance against lodging [74]. Together with miR529 and miR535, miR156 regulates SPLs that orchestrates differential organ development and structure. Overall the miR156-SPL, miR159-MYB33, and miR172-AP2 are involved in the regulation of leaf morphogenesis, floral organ development, root morphogenesis, and drought stress response, respectively [88,89,90]. Under drought, miR159a/b is down regulated resulting in an increase in MYB expression and reduction in growth. In addition, MYB has been shown to play a role in programmed cell death [79,91]. ARFs (10, 16, and 17) were regulated by miR160 [92] where when up regulated, miR160 reduced ARF (10 and 16) expression and produced shorter roots. However increased ARF16 activity resulted in reduced lateral root growth which is advantageous in energy conservation during drought [33,93]. ARF17 on the other hand inhibits adventitious root formation [94] (Figure 2).

Enlarged root and drought tolerance was observed in rice plants over-expressing OsNAC10 which results in higher yields [95]. The architectural changes to the roots are driven by miR166. Further, Zhang et al. (2018) provided molecular and genetic evidence that miR166 targets OsHB4 that regulates leaf morphology and vascular development [96]. Leaf rolling in drought exposed plants is a result of vascular constriction by miR166 on the xylem. In Acacia, miR166 was reported to regulate HD (Homeo Domain)-ZIP transcript genes for xylem development [97]. In addition, miR166 has been implicated in the development of leaf polarity by targeting HD-ZIP genes where their expression is differential in abaxial and adaxial regions of leaves [98]. The miR166 family has a greater tendency to regulate lateral root development under drought stress rather than under drought signaling. Further, miR166 via the posttranscriptional regulation of HD-ZIP results in cell development of roots, meristem, and leaves in *M. truncatula* [99]. Liu et al. (2007) reported that HD-ZIP III positively regulates lateral root formation [100]. However, when miR166 levels are elevated, HD-ZIP III is down regulated resulting in a reduction of lateral root formation under drought [99]. The knockdown of miRNA166 in rice led to morphological changes associated with drought tolerance such as leaf rolling and constriction of xylem [96] (Figure 2). This miRNA was either down or up regulated in roots and shoots in response to drought in different plant systems [10,101]. Together with miR167 and miR390, this protein acts on ARFs and controls root formation [33]. Additionally, during mineral deficiency in rice, miR167, miR394, and miR399 regulate physiological changes to the adventitious root growth in drought stress [102] (Figure 2).

Drought also affects the reproductive tissues in plants. Cheah et al. (2017) reported that four inflorescences specific miRNA, miR5485, miR5487, miR5492, and miR5517 which targets *SRK* (S-domain receptor kinase), *ARF*, *SPL11*, and KH (K homology) domain respectively and two non-inflorescences specific miRNA, miR169d, and miR169f.2 that targets *BAK1* (Brassinosteroid Insensitive 1-Associated Kinase I) and NFYA2. These targets mostly include flowering and embryogenic developmental genes under drought condition in rice [26]. miR5485, miR5487, miR5492, and miR5517 were not reported in other plant systems (miRbase). CBF/DREBs regulates the expression of miR169g, which in turn is induced in roots and shoots [103]. APETALA2 (*AP2*) genes that are involved in floral organ identity were also targeted by miR172 in response to both biotic and abiotic stresses [98]. Further, drought responsive novel miRNAs (n-006, n-192, n-002, n-063, n-137, n-024) have been reported in rice to target several important genes such as CK1 (CYCLIN-DEPENDENT KINASE INHIBITOR), *ELF* (EARLY FLOWERING PROTEIN), *GLUTAREDOXIN 2*, *GST* (GLUTATHIONE S-TRANSFERASE), *GPI-ANCHORED PROTEIN*, *OsFBX213* (F-box domain containing protein 213), and *SKP1-LIKE PROTEIN 1B*. These genes have roles in stress management in shoots, roots, and flowers. The expression profile analyses of these genes observed in vegetative and reproductive tissue may suggests possible functions for these genes in response to drought [104] (Figure 2).

## 4. The Role of miRNAs on Photosynthesis and Respiration during Drought

Drought suppresses photosynthetic activity and electron transport while enhancing respiration [105,106]. As a consequence of the above, drought reduces carbon dioxide fixation and starch storage in plants [107]. Therefore, maintaining a good rate of C-H synthesis is important to protect against drought in plants. As photosynthesis occurs in the leaf, leaf architecture is crucial in contributing to modulating photosynthesis and development. Leaf growth induces a tri-directional growth in plants directing the flow from source to distal, abaxial, and lateral tissues. In rice, the flag leaf is the location of photosynthates which is utilized for plant growth and development [108]. High levels of novel n-024 and n-063 were found in the flag leaf that targets GLUTATHIONE S-TRANSFERASE and EARLY FLOWERING PROTEIN respectively. Both these miRNAs and their targets are potential candidates for crop improvement [75]. In Arabidopsis, miR408 was also highly expressed in leaf tissues and is regulated by SPL7 and HY5 which is implicated in copper and light signaling. Their roles in copper and light is tied to copper apportioning to chloroplasts and higher levels of plantacyanin which is linked to light reaction of photosynthesis implicating a role for miR408 as a photosynthetic regulator in Arabidopsis [109]. In rice, miR408 cleaves OsUCL8 (plastocyanin-like protein) to positively regulate photosynthesis and grain yield [10]. The level of miR408 transcript is high in drought tolerant cultivars and low in drought sensitive lines [104].

The NFY transcription family are associated in the regulation of photosynthesis under drought stress where they are stipulated to work in concert with miR167 and miR169 [110] (Figure 2). In rice, miR397 is down regulated under drought [54,111] whereas miR397b is up regulated by ABA and drought in Arabidopsis. In tolerant soybean however, miR397a/b is down regulated during water stress conditions [112]. This transcript targets β-fructofuranosidase (FFase) and modulates metabolic activity of starch and sucrose in plants [113]. The fluctuation in the levels of miR397 expression (up or down regulation) regulates photosynthesis and respiration. In addition, miR397 targets laccase, which explains why reduced root growth is observed in dehydrated knockout mutants [114,115,116]. Yet another modulator, miR398, regulates respiration in rice by targeting cytochrome C oxidase subunit V (COX5b), which acts as an electron transporter in respiration [41,114]. However, further investigation is required to establish the functionality of miR398 in abiotic stress (Figure 2).

## 5. The Role of miRNAs in Stress Modulation

### 5.1. miRNAs and Senescence

**Figure 3:**
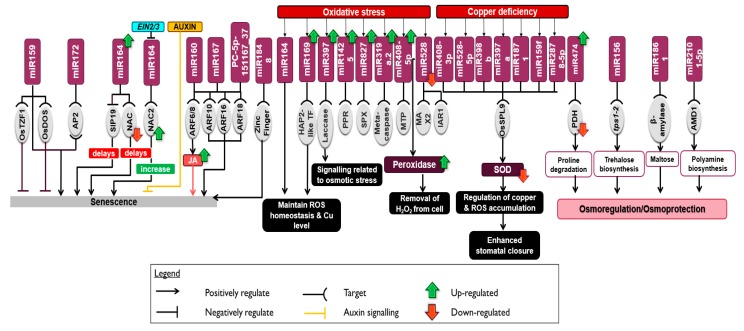
The regulation of microRNA under drought stress involving senescence, antioxidant defense and osmoregulation. The legend above provides elaboration on the functions of each arrow. The above miRNAs were identified under drought stress and their regulation resulted in a positive or negative effect on target transcription factors (ARF, NAC, ZF, HAP-2, SPL) or genes. These interactions resulted in either an increase or decrease in the expression of genes related to senescence, oxidative stress, and osmoprotection for better adaptation under drought.

Senescence effects are most clearly seen in the leaf and is a means by which plants moderate stress. We found that there are ABA regulated miRNA like miR172a, miR172c, and miR172d in leaves, which target AP2 transcription factors to regulate the process of senescence in rice. In senescence resistant cultivars these miRNAs are expressed at high levels. Two zinc finger transcription factors; OsTZF1 (CCCH-tandem zinc finger protein 1), and OsDOS (delay of the onset of senescence protein) that are targeted by miR159 negatively regulate senescence and therefore provide tolerance against abiotic stresses like drought in rice [116,117]. In addition to the Zn-finger factors, there are several ARFs (ARF8, 10, 16, 18) that regulate miR160a, miR167, and PC-5p-151167_37 in the regulation of senescence. ARF6 and ARF8 regulate the synthesis of jasmonate, which leads to increased expression of SAGs (Senescence Associated Genes) and elevated levels of JA (jasmonic acid) biosynthesis. This could be a possible mechanism used by miR167 to mediate senescence-resistance via JA signaling. Together with miR167 and miR172, miR159 mediates senescence resistance in rice [118]. Further, miR164 was highly expressed in rice leaves where it negatively regulates SIP19 (salicylic acid-induced protein 19), which delays senescence [119]. High levels of miR164a, miR164b, miR164d, and miR164e expression decreased NAC1 and NAC21/22 expression thus resulting in delayed leaf senescence in Arabidopsis [18]. NAC genes are also targeted by miR164 in rice to negatively regulate drought tolerance [70]. It is believed that miR164 uses this mechanism to regulate senescence in rice. Another regulator of senescence in rice is *EIN2*. Mutants of this gene showed delayed senescence in rice. It is believed that *EIN2* and *EIN3* inhibits miR164 and results in the induction of NAC2, which leads to elevated levels of senescence. Auxin on the other hand negatively regulates senescence in leaves [120]. While the process of senescence is poorly understood, key miRNAs involved in orchestrating the delay or hastening of senescence in rice are miR159, miR160, miR164, miR167, miR172, and miR1848. These miRNAs regulate leaf senescence through a well-orchestrated phytohormone signaling pathways [119] (Figure 3).

### 5.2. miRNAs in Antioxidant Defense

Reactive oxygen species (ROS) are produced in response to drought stress in various cellular compartments such as chloroplast and peroxisomes [121]. Elevated levels of ROS are potentially toxic to cells if not kept under check. Therefore, significant tolerance may be afforded by ROS detoxification in rice. Plants have evolved a defense mechanism that consists of low-molecular-weight antioxidants and antioxidative enzymes that facilitate ROS scavenging. Enzymes like superoxide dismutase (SOD), peroxidase (POD), catalase, glutathione reductase, and ascorbate peroxidase (APX) defend against these toxic radicals [122,123]. Li et al. (2011) in their study of oxidative stress in rice identified seven miRNAs responsible for the regulation of H_2_O_2_ stress (miR169, miR319a.2, miR397, miR408-5p, miR528, miR1425, miR827) that target HAP2-like transcription factor, metacaspase, laccase, MTP (Monosaccharide transport protein), MAX2 (F-box/LRR-repeat MAX2), and IAA-alanine resistance protein 1 (IAR1) like, PPR (Pentatricopeptide repeat protein) and SPX (SYG1/Pho81/XPR1 proteins) domain respectively [124]. All these miRNAs except miR528, miR319a.2, and miR408-5p, were up regulated in response to oxidative stress. miR319a.2 targets metacaspase, which is involved in programmed cell death in plants. Down regulation of miR528 resulted in increased SOD activity in rice. The same has been noted in other cereal crops like maize and wheat [39,125]. This therefore implies that miR528 is involved in detoxification during drought stress. In Arabidopsis however, miR398 was repressed and resulted in the up regulation of SOD [126] (Figure 3).

In a study between resistant Nagina and sensitive Pusa Basmati, miR164 and miR169 formed part of a regulatory node to maintain ROS homeostasis and rice copper levels through interplay of Cu-transporters, enzymes, and transcription factors [127]. Through copper mediated deficiency, certain cultivar specific miRNAs (miR159f, miR397a, miR398b, miR408-3p, miR528-5p, miR1871, and miR2878-5p) down regulated genes that are involved in the regulation of copper (e.g., SODs), ultimately leading to enhanced resistance towards ROS and stomatal closure in the resistant cultivars. This was achieved via the OsSPL9 transcription factor [17]. In drought a number of peroxidases are repressed in rice roots (LOC_Os05g04470.1, LOC_Os07g48030.1, LOC_Os07g48060.1, LOC_Os04g59260.1). In drought tolerant wheat, peroxidase levels were elevated in root cell walls. However, peroxidases act in contradiction. While peroxidases are essential for maintaining tolerance against drought stress, high levels of OH^−^ in cells may result in loss of structural integrity implying that a correct balance in peroxide levels is essential at managing stress [128,129] (Figure 3).

### 5.3. miRNA in Osmotic Stress during Drought

Plants also handle drought stress through the accumulation of osmoprotectants that are able to provide cell stability and turgor pressure [111,130]. Though these osmoregulators may vary from plant to plant, one osmolyte that plays a multifunctional role in defense is proline. Proline synthesis and degradation is responsible for the regulation of drought stress. Up regulation of miR474 has been referenced to control proline degradation in rice [54] by decreasing the expression of PDH (Pyruvate dehydrogenase), which results in improved drought tolerance. Trehalose, a non-reducing disaccharide sugar, is also associated to abiotic stress tolerance [131] through its ability to replace water molecules and rehydrate membranes and biomolecules [132,133]. miR156 was reported to be elevated in mutant Arabidopsis of trehalose-6-phosphate synthase (tps1-2) [134], a key enzyme for trehalose biosynthesis [135] (Figure 3).

Under drought stress, plants are conditioned to undergo lower photosynthetic activity. To support respiration in such conditions, starch degradation by β-amylase is required which increases the level of maltose that plays a role as an osmoprotectant [136]. Based on degradome data, miR1861 was found to target a transcript-encoding β-amylase (Os10g32810) in rice [137]. Polyamines, which are osmoprotectants, were implicated in drought stress tolerance [138]. Target mRNAs of miR2101-5p encoding an S-adenosylmethionine decarboxylase proenzyme (AMD1) is an important enzyme for polyamine synthesis in plants [139].

In an in silico study conducted by Umate and Tuteja (2010), 12 miRNAs that targeted helicase genes in rice were identified [140]. Out of these 12, miR164, miR408, and miR414 were down regulated and known to affect DEAD-box helicases (DBH) that play a role in up regulating the response to drought and salinity stress in plants [141]. miR156, miR159, and miR396 are highly regulated in Arabidopsis however the same miRNAs are severely down regulated in rice [33]. Further, a DUF1645 domain containing OsSGL (Stress tolerance and Grain Length) protein in Arabidopsis and an overexpressing rice lines of the same exhibited tolerance to osmotic stresses through accumulation of proline. In addition, when RNA-Seq analysis was conducted on the overexpressing rice lines, an abundance of stress related genes were found at enhanced levels where scavenging enzymes for ROS were among the most dominant [142]. A differential expression profiling study on drought leaves at vegetative stage conducted by Cheah et al. (2015) revealed that miR397a/b targets osmotic stress-activated protein kinase and were found to be up regulated in IR64 while down regulated in Vandana and Aday Sel [114] (Figure 3).

## 6. Differences and Similarities in Drought Response between Rice and Other Plant Systems

**Figure 4:**
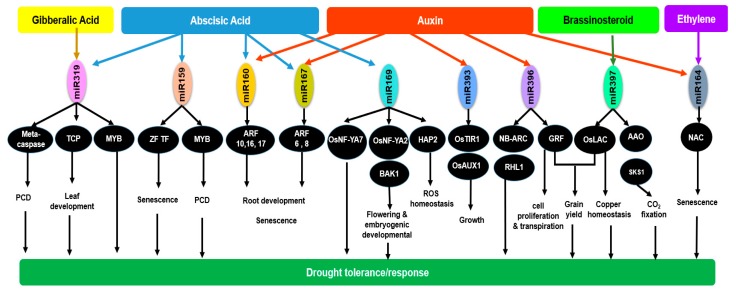
The regulation of drought tolerance and response in rice. The regulation of drought tolerance and response by key miRNAs via auxin, ABA, GA, BR, and ethylene. Processes like programmed cell death, senescence, ROS homeostasis, growth and development, photosynthesis, and yield are regulated by miRNAs through its respective targets for better drought tolerance. The colored arrows show the regulation of miRNA by different phytohormones (yellow: GA, blue: ABA, orange: auxin, green: BR, purple: ethylene). The black arrows indicate the regulation of target genes/transcription factors by miRNA and the regulation of drought responses by the target genes/transcription factors. The black ovals represent target genes/transcription factors).

Based on the exhaustive information provided in this review, key conserved miRNAs that play an important role in drought stress in rice and other plant systems (miR160, miR164, miR167, miR169, miR319, miR393, miR396, and miR397) and their regulation thereof of various biological processes is summarized in Figure 4 [39,47,76,104,143,144]. As depicted in Figure 4, all these processes, which include programmed cell death, senescence, ROS homeostasis, growth and development, photosynthesis, and yield are interrelated and are required for the survival and well-being of plants. In the following paragraphs we elaborate on the similarities and differences observed between rice and other plant systems in responding to drought based on these key miRNAs.

### 6.1. Hormones Are Central to Drought Regulation

As anticipated, in both rice and other plant system the various physiological processes affected by drought are tightly regulated by an interplay between miRNA and hormones. In plants, hormones play an integral role in drought stress regulation and response. Accordingly, Figure 4 shows that rice, and in general plants, mitigate drought response through cross talk and interplay between ABA, IAA, ETH, and BR. While all these hormones interact to alleviate the stress induced by drought, ABA is the most crucial hormone for drought stress regulation followed by auxin and ethylene. In Section 2.1 and Section 2.2 it is stated that the increase and decrease in the levels of hormones are tightly linked to their miRNAs targets (Figure 4) [36]. However, there are miRNAs that are regulated in an ABA independent manner and certain miRNAs with no reported hormone targets (Figure 4). These miRNAs require further study to determine if hormones or signal molecules play a role in their regulation. We also provided an in-depth overview on the miRNAs-Transcription Factors-genes involved in the hormonal regulation of root and shoot growth, photosynthesis, respiration, and stress modulation (Section 2, Section 3, Section 4 and Section 5; Figure 1, Figure 2, Figure 3 and Figure 4). Hormone associated regulation of miRNAs, target genes, and transcription factors modulate plant vegetative and reproductive development. In drought resistant plants, a proper regulation of these hormone-driven processes by miRNAs results in its survivability and productivity. The opposite is observed in drought susceptible plants. The changes to the structure of the plants to adapt to drought stress will be elaborated in the following section. However, one interesting finding from analyzing the involvement of miRNAs response to hormones in plants is that similar miRNAs may either be up or down regulated in different plant systems due to variation in developmental stages, growth conditions, or stresses applied. These miRNAs seem to work in pairs where there are sets of miRNAs that are up regulated while others are down regulated implying a possible feedback regulation in most processes. For example, when miR167 and miR169 were down regulated, miR319 was up regulated in response to ABA in rice under normal conditions. Nonetheless, though the role of hormone in regulation of drought stress is well-defined, functional analyses of these target miRNAs are required to provide a clear picture on how these biological processes are fine-tuned by these conserved and novel miRNAs.

### 6.2. The Semi-Aquatic Rice Versus Terrestrial Plants

Further through our review at the miRNA level, we are able to observe some similarities and differences between rice and terrestrial plants in their response towards drought stress. Being a semi-aquatic plant, rice requires constant water supply making it more sensitive to drought as compared to other terrestrial crops which are better adapted to thrive under water deficit conditions. Root architecture plays an important role in drought tolerance and may differ according to plant systems. As a monocotyledonous plant, rice has fibrous root systems which is different from that of the dicotyledonous Arabidopsis with tap roots. Arabidopsis is more likely to be resistant to drought than rice, as taproot enables the plant to reach for deep underground water. miR160 and miR167 are two miRNAs that play an important role in root architecture control via auxin. In severe drought conditions, plants are generally designed to save all energy avoiding processes that are not relevant to its survival. One such process in rice is the adventitious root formation under water deficit. The down regulation of miR160 in rice under drought condition contributes to reduced lateral root growth and inhibits adventitious root formation in both rice and Arabidopsis. However, miR160 was differentially expressed in various studies conducted on wheat, implying that the root growth regulation via auxin may be modulated by the level of stress imposed. Meanwhile, the inhibition of adventitious root formation induced by the down regulation of miR167 under drought in rice is in contrast with Arabidopsis and wheat implying that under stress conditions root growth regulation by miR167 is different in rice as compared to Arabidopsis and wheat. Hence inhibition of adventitious root and reduced lateral root is vital for efficient expenditure of energy, water use, and generating tolerance towards a stressful environment [5] (Figure 2; Figure 4; Section 3).

Plants experience oxidative stress and senescence followed by the accumulation of osmoprotectants under drought stress. The regulation of these processes seem to be conserved across all plant systems. miR169 was up regulated under oxidative [124] and drought stress [90] to slow down respiration and cell differentiation. Accumulation of ROS is one of the earliest events observed upon drought stress. Hence, miR169 helps in scavenging the excessive ROS to alleviate the negative effects of drought stress. In addition, copper plays an important role in maintaining ROS homeostasis. In copper deficient conditions, miR408, miR528, miR398, miR397, miR1871, miR159, and miR2878 were up regulated which increased the ROS accumulation resulting in stomatal closure. Through stomatal closure, respiration is reduced and thus results in increased photosynthesis. This results in an increase towards yield through the activation of miRNAs such as miR408 and miR397. Apart from that, miRNA that targets senescence related proteins and transcription factors are up regulated to delay senescence to reduce yield loss with decrased drought tolerance. For example, miR164 was up regulated for delayed senescence and targets NAC that negatively regulates drought tolerance. miRNA that are involved in the synthesis of osmoprotectants, such as proline and trehalose, were also induced, indicating that the plants are programmed to protect against cellular and molecular harm. Jointly, the regulation of miRNA helps to mitigate stresses induced by drought. Overall, this implies that the above stress responses which are important for plants survival and homeostasis, are conserved across all plant systems [145,146].

Under severe drought conditions, stomata may remain closed completely depending on the plant species. The stomata may be intermittently opened and closed in tolerant plant species for processes such as photosynthesis and carbon fixation [147]. Stomatal movement is usually regulated by ABA to restrict water loss through transpiration via the interaction between miR169 and MYB. The down regulation of miR169 in Arabidopsis under drought stress enhances stomatal closure. However, miR169 was up regulated in rice and differentially expressed in wheat to confer drought tolerance. This indicates that rice may be less tolerant to drought as compared to Arabidopsis which has better stomatal aperture control which prevents excessive water loss through respiration and transpiration [147]. Other than changes in stomata, there are other observable changes such as leaf rolling in drought stressed plants. Leaf rolling is also one of the earliest signs observed in plant under drought stress which serves as an adaptive response to prevent water loss. The knockdown of miR166 in rice is associated with leaf development and leaf rolling under drought stress. miR166 was also down regulated in drought resistant wheat [143]. However, the suppression of miR166 in Arabidopsis resulted in other developmental defects and late flowering with good tolerance against drought [148]. Besides, similar miRNAs in different plant systems may regulate different organs under drought. For example, down regulation of miR393 represses GRF activity, lowers cell proliferation, and reduces transpiration in rice. In Arabidopsis, however, the up regulated miR393 cleaves auxin related genes leading to the inhibition of lateral root growth via ABA, while resulting in differential expression in wheat without any clear function [38] (Figure 4). This shows the versatility of some of the miRNA players in drought response and regulation.

Further, drought stress can affect grain yield as reproductive processes and grain filling are disrupted due to lack of resources which directs the plant to undergo early senescence where the duration of seed-filling is reduced and the translocation of assimilates from source to sink is enhanced [144]. The induction of miR397 under normal conditions contributes to higher grain yield and panicle branching through their positive involvement in photosynthesis (Figure 2; Figure 4; Section 4). However, under drought, miR397 is down regulated in rice and up regulated in Arabidopsis and wheat. This implies that rice is more sensitive to water deficit and tends to retard growth as part of its programmed response to conserve energy and survive. Flag leaf is a vital part of the plant that provides photosynthates for panicles and carbohydrate for grain filling [107]. In addition to the conserved miR397, two novel miRNAs namely n-024 and n-063 were significantly induced in flag leaf during drought in rice [103]. These novel miRNAs were also implicated in rice yield and not reported in other plant systems (Figure 2).

## 7. Conclusions and Future Prospects

This review summarizes findings regarding miRNAs and their versatile function in targeting genes and transcription factors to repress the effect of drought in rice. The information is compared with reported findings from other plant systems. It appears that these miRNAs are part of a regulatory network that control metabolic pathways in response to drought stress, highlighting their functional role in drought tolerance or drought avoidance. In particular, these modulators control vital processes in plants including growth, development, photosynthesis, respiration, and other drought-induced stresses [149].

In the recent years, a vast number of drought-responsive miRNAs have been discovered using NGS [150] and their functional analyses provide a better understanding of mechanisms by which plants counter this stress. However, since a multitude of drought-responsive miRNAs have been observed in rice and other plant species, further research on their functions, signaling pathways, and gene networks is required for a better understanding of the role played by miRNAs in drought. In addition, further exploration of regulatory pathways upstream of miRNA and downstream of the target genes will increase our understanding of drought stress adaptation in rice and other plant systems.

Globally, from the various studies conducted at the miRNA level in different plant systems, miRNAs appear to be involved in the regulation and expression of multiple target genes. There is also evidence of cross talk between processes and pathways associated with drought stress response in rice. Here we are able to conclude that miRNAs provide a link between environment and plant development based on miRNA-mediated response to drought stress. These modulators have great potential for development of resistant varieties, with improved agronomic traits through next generation technologies and genetic engineering.

Through a systematic review of literature on the role of miRNAs in drought stress modulation in rice and other plant systems, we have identified a few miRNAs that have potential for manipulation and use in crop improvement. As observed in drought sensitive plants, stress retards growth and development and this has a direct consequence on yield [21,31,33,62]. Therefore, it is crucial to identify key players of growth and development that can be used in breeding for drought tolerant plants. The growth and developmental process is tightly linked to the photosynthetic ability of the plant where, when affected, it interferes with its source and sink ability. This results in changes in growth such as reduced lateral root formation as well as enlarged root, affected flowering stages, low seed setting ability, and overall lower yield. miR408, miR156, miR167, miR393, miR396, miR1432, miR444, miR172, and miR397 have been reported to influence growth and yield in rice and other plant systems. Additionally, miRNAs such as miR156, miR159, miR160, miR164, miR167, miR169, miR319, miR396, and miR397 have been reported to reduce negative effects of drought through regulation of hormones, development, physiology, and stress in rice (Figure 1, Figure 2, Figure 3 and Figure 4). As these miRNAs play a role in regulating multiple processes, further dissection of these miRNAs is crucial for the development of drought resistant rice [151,152].
